# Prognostic value of low-density lipoprotein cholesterol in IgA nephropathy and establishment of nomogram model

**DOI:** 10.3389/fendo.2023.1037773

**Published:** 2023-02-10

**Authors:** Zhang-Yu Tian, Ai-Mei Li, Ling Chu, Jing Hu, Xian Xie, Hao Zhang

**Affiliations:** ^1^ Department of Nephrology, The Third Xiangya Hospital, Central South University, Changsha, Hunan, China; ^2^ Department of Pathology, The Third Xiangya Hospital, Central South University, Changsha, Hunan, China

**Keywords:** IgA nephropathy, low-density lipoprotein cholesterol, prognosis, ESRD, nomogram prognostic model

## Abstract

**Background:**

Dyslipidemia is closely related to kidney disease. We aimed to investigate the relationship between low-density lipoprotein cholesterol (LDL-C) and prognosis of IgA nephropathy (IgAN) and build a nomogram prognostic model.

**Methods:**

519 IgAN patients with 61 months median follow-up were enrolled and divided into two groups based on the cut-off value of baseline LDL-C (2.60 mmol/L): the high group (n=253) and the low group (n=266). Renal survival was assessed by Kaplan⁃Meier (KM) survival curve. Risk factors were identified by COX regression analysis. The area under the receiver operating characteristic (ROC) curves (AUC), concordance index (C-index), and calibration curves were applied to evaluate the nomogram model.

**Results:**

KM survival curve analysis showed that the high LDL-C group had worse renal survival than the low LDL-C group (χ2 = 8.555, p=0.003). After adjusting for confounding factors, Cox regression analysis showed the baseline LDL-C level was an independent risk factor of end-stage renal disease (ESRD) in IgAN (HR=3.135, 95% CI 1.240~7.926, p =0.016). LDL-C, segmental sclerosis, tubular atrophy/interstitial fibrosis, the prevalence of cardiovascular disease, 24-hour proteinuria were identified and entered into the nomogram models, with AUC of 0.864, 0.827, and 0.792 respectively to predict the 5-, 8-, and 10-year risk of ESRD in IgAN. The C-index of this prediction model was respectively 0.862, 0.838, and 0.800 and was well-calibrated.

**Conclusion:**

Elevated LDL-C level is a predictive factor for the prognosis of IgAN. We developed a nomogram model that can predict the risk of ESRD in IgAN by using LDL-C ≥ 2.60 mmol/L.

## 1 Introduction

Immunoglobulin A nephropathy (IgAN) is the most common primary glomerulonephritis worldwide, and approximately 30% of IgAN patients develop ESRD within 20 years (1, 2). Recognizing risk factors of end-stage renal disease (ESRD) and establishing a nomogram prognostic model for patients with IgAN is worthwhile.

Guidelines for lipid management of chronic kidney disease (CKD), particularly in ESRD is inconsistent worldwide at present (3).The KDIGO guidelines focus on cardiovascular risk to guide treatment and do not recommend treating any patient based on “high” cholesterol levels per se. However, the 2016 European Guidelines suggest most patients with a 10-year cardiovascular risk of 5-10% would benefit from lipid-lowering therapy if their baseline LDL cholesterol was 2.6-4 mmol/l (4). KDIGO guidelines recommend that dialysis patients should not be started on statins or a combination of statins and ezetimibe (5). The 2016 Canadian Cardiovascular Society Guidelines for the Management of Dyslipidemia recommend that patients on dialysis should begin treatment if they are likely to remain on dialysis for many years or receive a transplant (6).

There is a strong, robust and graded association between LDL-C levels and cardiovascular risk, and lowering LDL-C reduces cardiovascular risk in a dose-dependent manner (7, 8). Furthermore, accumulation and lipotoxicity can lead to glomerular podocyte and proximal tubular epithelial cell dysfunction (3, 9). Study showed hypertriglyceridemia was prevalent in CKD patients, and it was independent risk factor for moderate tubular atrophy/interstitial fibrosis (10). Oxidized LDL-C (oxLDL) can promote glomerulosclerosis through infiltration of monocytes/macrophages and overexpression of adhesion molecules (11). Levels of lipids are closely related to renal function, and lipid metabolism is often disturbed in IgAN patients with predominantly nephrotic syndrome (12, 13). Hypercholesterolemia and hypertriglyceridemia are reported to be relevant to the deterioration of renal function in adults with IgAN (14). However, the relationship between levels of LDL and prognosis of IgAN was not yet studied.

The utilization of lipid-lowering agents in early kidney disease in the 2013 KDIGO guidelines are relatively conservative (3). Assessment of lipids level in renal injury and prognosis of IgAN is currently undervalued. The aim of this study was to investigate the prognostic relevance of LDL-C levels in IgAN patients, with the expectation of informing LDL-C treatment targets in lipid management of IgAN.

## 2 Method

### 2.1 Patients selection

**Figure 1:**
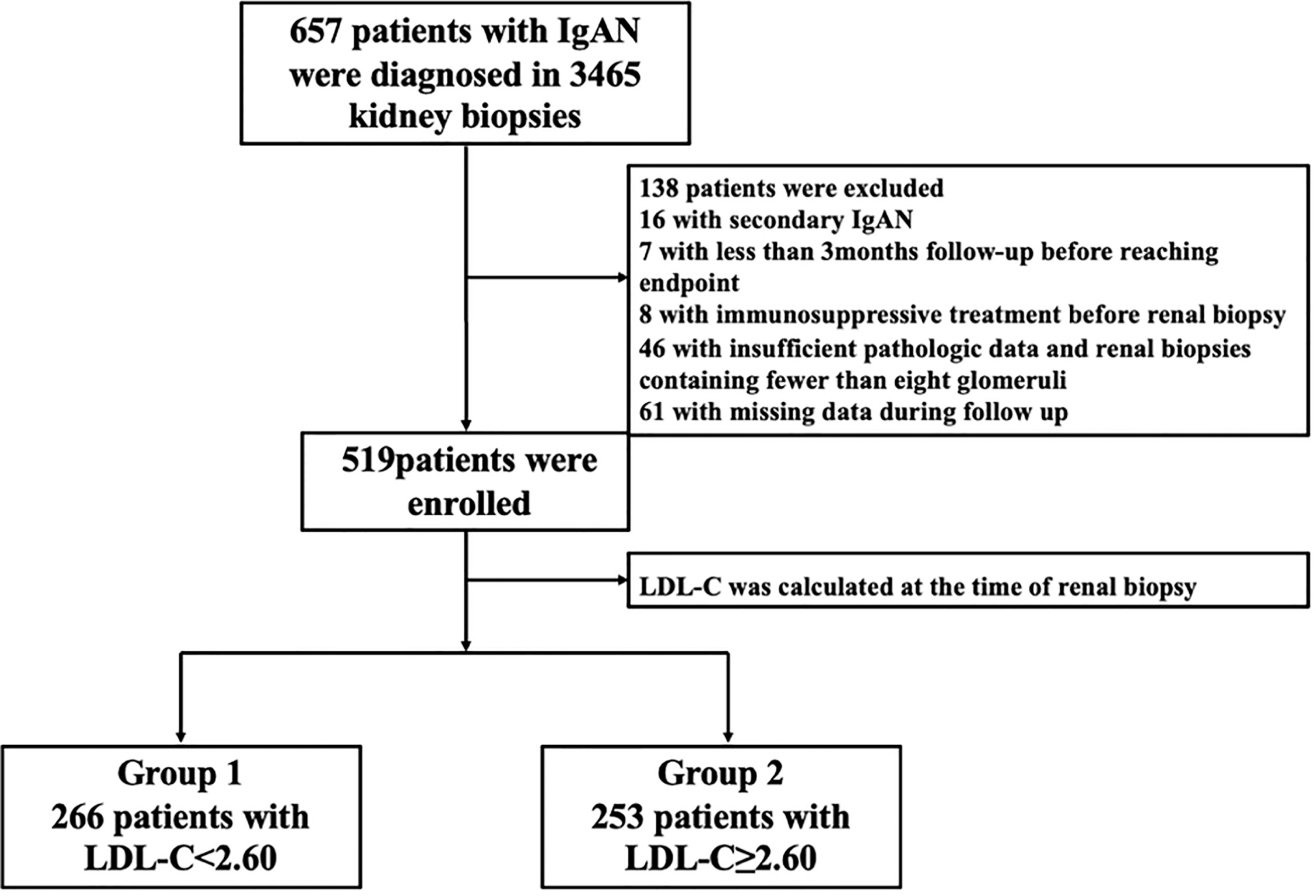
The selection process for patients in a flow chart. IgAN, IgA nephropathy; LDL-C, low-density lipoprotein cholesterol.

As shown in a flow chart (**Figure 1**), there were 3465 patients who underwent renal puncture biopsy between April 13, 2012 and April 29, 2022, and 657 IgAN patients were automatically screened from the electronic medical record system of the Third Xiangya Hospital of Central South University. The key inclusion criteria were primary IgA nephropathy confirmed on biopsy; estimated glomerular filtration rate (eGFR)>15 ml·min-1· (1.73 m2)-1 at the time of renal biopsy; renal tissue specimens with at least 5 mm cortical and 8 glomeruli. Major exclusion criteria were combination of other types of primary glomerular disease, or secondary IgAN, or systemic disease (e.g., systemic lupus erythematosus, severe infection, etc.); immunosuppressive therapy before renal biopsy; incomplete clinical or pathological data; follow-up<3 months. After reviewing the electronic medical records, 16 patients with secondary IgAN, 7 patients with less than 3 months follow-up before reaching endpoint, 8 patients with immunosuppressive therapy before renal biopsy, 46 patients with less than 8 glomeruli, and 61 patients with incomplete data were excluded. Finally, 519 patients were included in this study. The cut-off value of the baseline LDL-C of 519 IgAN patients were divided into two groups: the high group (LDL-C ≥ 2.60 mmol/L, n=253) and the low group (LDL-C<2.60 mmol/L, n=266).

### 2.2 Ethical approval

This study was approved by the Ethics Committee of the Third Xiangya Hospital of Central South University (NO.22146)

### 2.3 Data collection

Age, sex, systolic blood pressure (SBP), diastolic blood pressure (DBP), cardiovascular disease (CVD), diabetes, serum albumin, serum creatinine (Scr), serum uric acid (SUA), blood urea nitrogen (BUN), estimated glomerular filtration rate (eGFR), triglyceride (TG), total cholesterol (TC), 24-hour proteinuria, pathological findings, and treatment were collected from the enrolled patients. Patients were followed up every 3 months with a follow-up deadline of July 27, 2022.

### 2.4 Relevant definitions

eGFR: Calculated by the MDRD formula recommended by the 2002 National Kidney Foundation ⁃ Guidelines for quality of survival in patients with kidney disease (8). eGFR [ml·min-1·(1.73m2)-1] =186.3× [Scr (mg/dl)]-1.154×[age (years)]-0.203× 0.742 (female).

Renal pathology: Pathological diagnosis was based on the IgAN Oxford Classification (8): (i) mesangial hypercellularity (M); (ii) endocapillary cellularity (E); (iii) segmental sclerosis (S); (iv) tubular atrophy/interstitial fibrosis (T); (v) crescents (C).

CVD: Including atherosclerosis, hypertension, myocardial infarction, and stroke.

Treatment: Treatment with glucocorticoids and/or immunosuppressants, including oral methylprednisolone or equivalent doses of prednisone for at least 3 months, and/or oral tacrolimus or mycophenolate mofetil treatment for at least 3 months.

Renal outcome: The progression to ESRD, defined by commencement of renal replacement therapy or an eGFR<15 ml·min-1· (1.73 m2)-1.

### 2.5 Statistics

The discriminatory power of various predictive factors for development of renal survival was tested by the area under the receiver operating characteristic curve (AUROC), and the optimal cut-off point of the LDL-C was obtained by calculating the Youden index. The Youden index is a method of evaluating the authenticity of a screening test, which represents the total ability of the screening method to detect true patients and nonpatients. A higher index is associated with a better effect and greater authenticity of the screening test. The LDL-C that corresponded to the maximum Youden index was then determined to be the optimal cut-off LDL-C in this study.

Statistical analyses were performed using SPSS 26.0 software and R statistical software. The Kolmogorov-Smirnov normality test was used to estimate the data distribution. Normally distributed data were expressed as mean ± standard deviation ( $\bar{x}$ ± SD) and one-way ANOVA was used for comparison between groups. Non-normally distributed data were expressed as median (interquartile range) and non-parametric tests were used for comparisons between groups. The chi-square test was used to analyze the count data. p<0.05 indicates statistical significance. Univariate COX regression analysis was performed to identify risk factors for IgAN. Risk factors with p values<0.05 in the univariate analysis were included in the multivariate cox regression. Multivariate cox regression was performed to identify independent risk factors, and a nomogram prediction model was developed using a stepwise approach to identify useful combinations of factors to predict IgAN prognosis. The performance of the column line graphs was assessed using the C-index and calibration plots with bootstrap samples. The C-index is a numerical measure of discriminative ability and the calibration curve is a graphical assessment of predictive ability that compare observed probabilities with nomogram-predicted probabilities. The column line graphs are constructed using the “rms” package.

## 3 Results

### 3.1 Patients’ general characteristics

519 IgAN patients were enrolled in this study, and the baseline characteristics of IgAN patients at the time of renal biopsy are shown in **Table 1**. The median age of IgAN patients was 32 (25, 42) years old, and 85.6% of patients were in CKD1-2 stage. Compared with patients in the low LDL-C group, patients in the high LDL-C group had lower baseline serum albumin (p<0.05) and higher age, male prevalence, incidence of CVD, 24-hour proteinuria, SUN, SUA, TG, TC (all p<0.05). In the IgAN Oxford Classification, the differences in M1, E1, S1, T1-2 and C1-2 prevalence were not statistically significant. But LDL-C is associated with IgA deposition (p<0.05). In terms of treatment, patients in the high LDL-C group received higher glucocorticosteroid (p<0.001) and immunosuppressants (p<0.05) compared with the low LDL-C group, but the difference between the two groups receiving RAS inhibitors, calcium channel blocker was not statistically significant. In conclusion, patients in the high LDL-C group had worse nutritional status, more pronounced proteinuria, more incidence of CVD, higher IgA deposition, poorer renal function and other clinical indicators at compared to patients with lower LDL-C.

**Table 1:** Demographic and clinicopathological characteristics of patients with IgAN.

Characteristic	Median (IQR) (n=519)	Low LDL-C group<2.60 (n=266)	High LDL-C group≥2.60 (n=253)	t/Z/x2	P value
*Age (years)	32 (25,42)	31 (24,39)	35 (26,46)	-3.094	0.002^#^
Sex (male,%)	253 (48.7)	117 (46.2)	136 (53.8)	4.954	0.026^#^
SBP (mmHg, $\bar{x}$ ±s)	120.63 ± 28.12	119.86 ± 30.453	121.43 ± 25.480	-0.634	0.526
DBP (mmHg, $\bar{x}$ ±s)	77.29 ± 19.03	76.74 ± 20.259	77.88 ± 17.674	-0.680	0.497
CVD (%)					
Prevalence of CVD	104 (20.0)	51 (49.0)	53 (51.0)	0.255	0.613
Incidence of CVD	49 (9.44)	18 (36.7)	31 (63.3)	4.565	0.033^#^
Diabetes (%)	15 (2.9)	10 (66.7)	5 (33.3)	1.469	0.226
*eGFR[ml·min-1· (1.73m2)-1]	91.70 (72.17,113.23)	95.67 (75.96, 112.06)	88.19 (68.59,116.17)	-1.583	0.113
CKD Classification (%)				12.84	0.005^#^
CKD1	276 (53.2)	154 (55.8)	122 (44.2)		
CKD2	168 (32.4)	81 (48.2)	87 (51.8)		
CKD3	66 (12.7)	31 (47.0)	35 (53.0)		
CKD4	9 (1.7)	0 (0.00)	9 (100.00)		
* 24-hour proteinuria (mg/d)	898.0 (324.0,2326.0)	606.5 (256.0,1289.0)	1663.5 (542.5,3233.0)	-7.339	<0.001^#^
* Scr (μmol/L)	78 (63,98)	77 (62,94)	79 (64,102)	-1.867	0.062
SUA (μmol/L, $\bar{x}$ ±s)	359.55 ± 112.56	346.16 ± 104.74	373.63 ± 118.83	-2.797	0.005^#^
*SUN (mmol/L)	4.87 (3.95,6.02)	4.76 (3.81,5.81)	5.00 (4.09,6.18)	-1.967	0.049^#^
*ALB (g/L)	37.70 (32.60,41.50)	39.20 (35.90,42.20)	34.10 (24.00,39.85)	-7.853	<0.001^#^
*TG (mmol/L)	1.36 (0.96,2.09)	1.12 (0.82,1.84)	1.67 (1.14,2.32)	-6.027	<0.001^#^
*TC (mmol/L)	4.86 (4.09,6.05)	4.12 (3.63,4.60)	5.92 (5.21,7.52)	-17.277	<0.001^#^
Oxford Classification (%)					
M1	161 (31.0)	75 (46.6)	86 (53.4)	2.036	0.154
E1	22 (4.2)	12 (54.5)	10 (48.9)	0.100	0.752
S1	147 (28.3)	79 (53.7)	68 (46.3)	0.509	0.476
T1-2	55 (10.6)	24 (43.6)	31 (56.4)	1.428	0.232
C1-2	59 (11.3)	29 (49.2)	30 (50.8)	0.117	0.732
IgA deposition (%)				10.488	0.033^#^
_	4 (0.8)	1 (25.0)	3 (75.0)		
+	116 (22.4)	52 (44.8)	64 (55.2)		
++	131 (25.2)	59 (45.0)	72 (55.0)		
+++	267 (51.4)	154 (42.3)	113 (57.7)		
++++	1 (0.2)	0 (0.0)	1 (100.0)		
Treatment (%)					
ACEI or ARB agents	334 (64.4)	164 (49.1)	170 (50.9)	1.735	0.188
CCB	79 (15.2)	35 (44.3)	44 (55.7)	1.801	0.180
Glucocorticosteroid	205 (39.5)	80 (39.0)	125 (61.0)	20.279	<0.001^#^
Immunosuppressant	145 (27.9)	59 (40.7)	86 (59.3)	8.986	0.003^#^

“*” indicates that data were not normally distributed and are expressed as median (interquartile range), M (Q). “#” indicates the difference was statistically significant (p<0.05). eGFR, estimated glomerular filtration rate; LDL-C, low-density lipoprotein cholesterol; SBP, systolic blood pressure; DBP, diastolic blood pressure; CVD, cardiovascular disease; Scr, serum creatinine; SUA, serum uric acid; BUN, blood urea nitrogen; TG, triglycerides; TC, total cholesterol; CCB, calcium channel blocker; IQR, interquartile range. -, +, ++, +++ and ++++ represent the intensity of IgA deposition in the mesangial membrane in immunohistochemistry.

### 3.2 Relationship between LDL-C and the prognosis of IgAN

**Figure 2:**
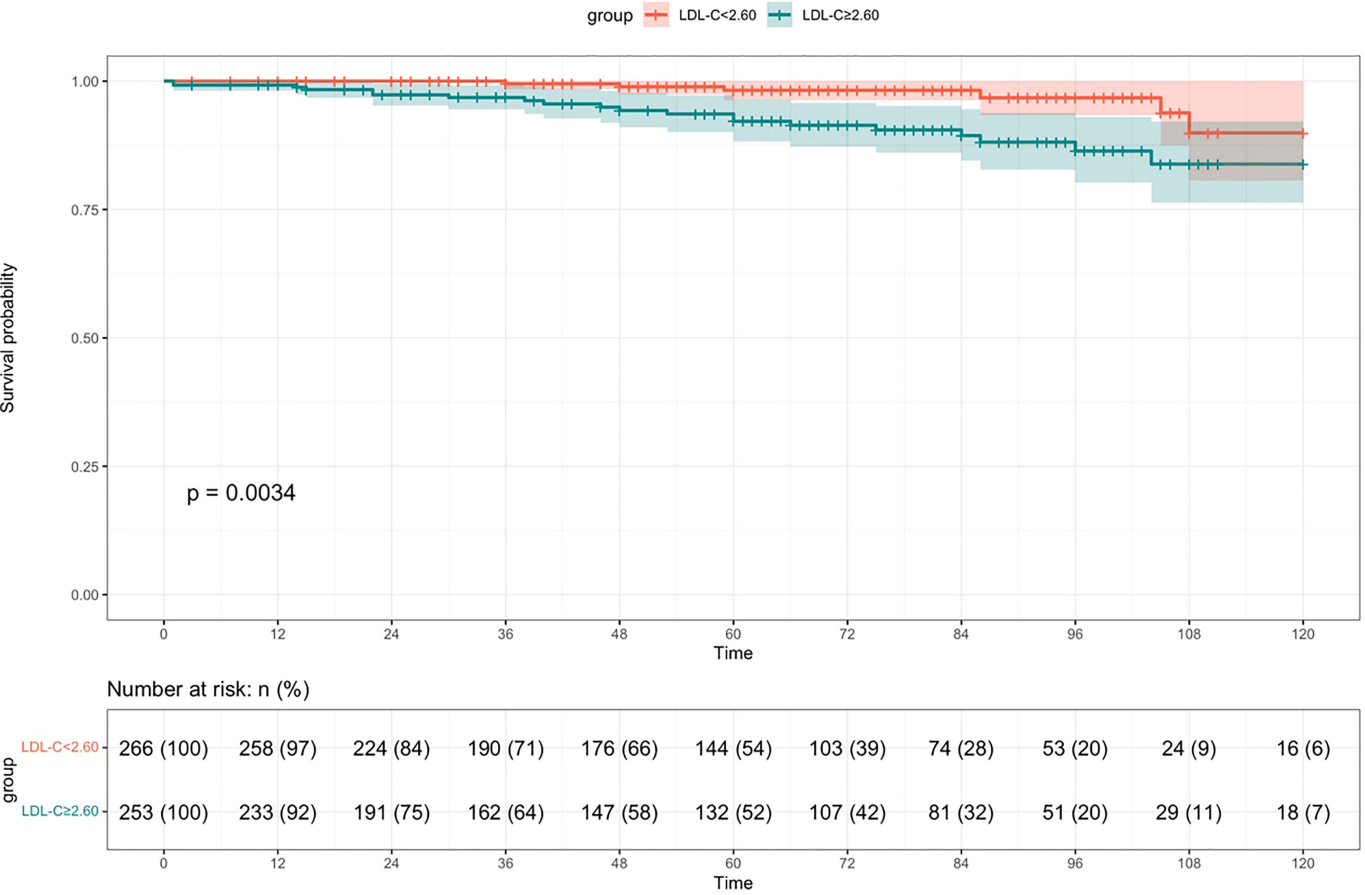
Survival rate of patients in the high LDL-C group compared to the low LDL-C group with IgAN (Kaplan-Meier survival curve).

During a median follow-up time of 61 (31,89) months, 26 cases were into ESRD. There were 20 cases of ESRD events in the high LDL-C group (76.92%), including 18 cases of renal dialysis, 2 cases of eGFR<15ml·min-1· (1.73 m2)-1. There were 6 endpoint events in the low LDL-C group (23.08%), including 5 cases of renal dialysis, 1cases of eGFR<15ml·min-1· (1.73 m2)-1. Kaplan-Meier survival curve analysis showed that renal survival in the high LDL-C group was significantly lower than in the low LDL-C group (χ2 = 8.555, p =0.003) (**Figure 2**). LDL-C was included in the Cox regression equation as a categorical independent variable. The univariate Cox analysis showed that high LDL-C, prevalence of CVD, SUA, BUN, Scr, 24-hour proteinuria, M1, S1, T1-2, and C1-2 were influential factors of prognosis of patients with IgAN (p< 0.05). Multivariate Cox analysis showed that LDL-C was an independent risk factor for renal prognosis in patients with IgAN (HR=3.135, 95% CI 1.240~7.926, p =0.016), suggesting that patients in the high LDL-C group had a 3.135-fold higher risk than the low group to enter ESRD. In addition to LDL-C, prevalence of CVD, S, T and 24-hour proteinuria were independent risk factors for IgAN in this COX model (p<0.05) (**Table 2**).

**Table 2:** Analysis of factors influencing poor renal prognosis in patients with IgAN (Cox regression equation).

Independent variables	Univariate analysis		Multivariate analysis	
	HR (95%CI)	P Value	HR (95%CI)	P Value
High LDL-C group	3.576 (1.435~8.911)	0.006^#^	3.135 (1.240~7.926)	0.016^#^
Female	1.481 (0.684~3.206)	0.319		
Age	1.022 (0.990~1.055)	0.175		
Prevalence of CVD	3.758 (1.735~8.140)	0.001^#^	3.956 (1.744~8.969)	0.001^#^
SUA	1.004 (1.001~1.007)	0.005^#^		
Diabetes	2.371 (0.560~10.050)	0.241		
BUN	1.183 (1.113~1.256)	<0.001^#^		
SCr	1.019 (1.014~0.103)	<0.001^#^		
ALB	0.969 (0.930~1.010)	0.134		
24-hour proteinuria	1.000 (1.000~1.000)	<0.001^#^	1.000 (1.000~1.000)	0.007^#^
M1	3.581 (1.652~7.761)	0.001^#^		
E1	3.548 (1.049~11.998)	0.080		
S1	2.900 (1.330~6.320)	0.007^#^	2.755 (1.219~6.224)	0.015^#^
T1-2	8.340 (3.864~18.003)	<0.001^#^	6.033 (2.716~13.400)	<0.001^#^
C1-2	3.988 (1.733~9.177)	0.001^#^		
Glucocorticosteroid	0.470 (0.213~1.037)	0.061		
Immunosuppressant	0.603 (0.273~1.333)	0.211		

LDL-C, low-density lipoprotein cholesterol; CVD, cardiovascular disease; Scr, serum creatinine; SUA, serum uric acid; BUN, blood urea nitrogen; HR, hazard ratio; CI, confidence interval.“#” indicates the difference was statistically significant (p<0.05).

### 3.3 Establish and validate the nomogram prognostic model

**Figure 3:**
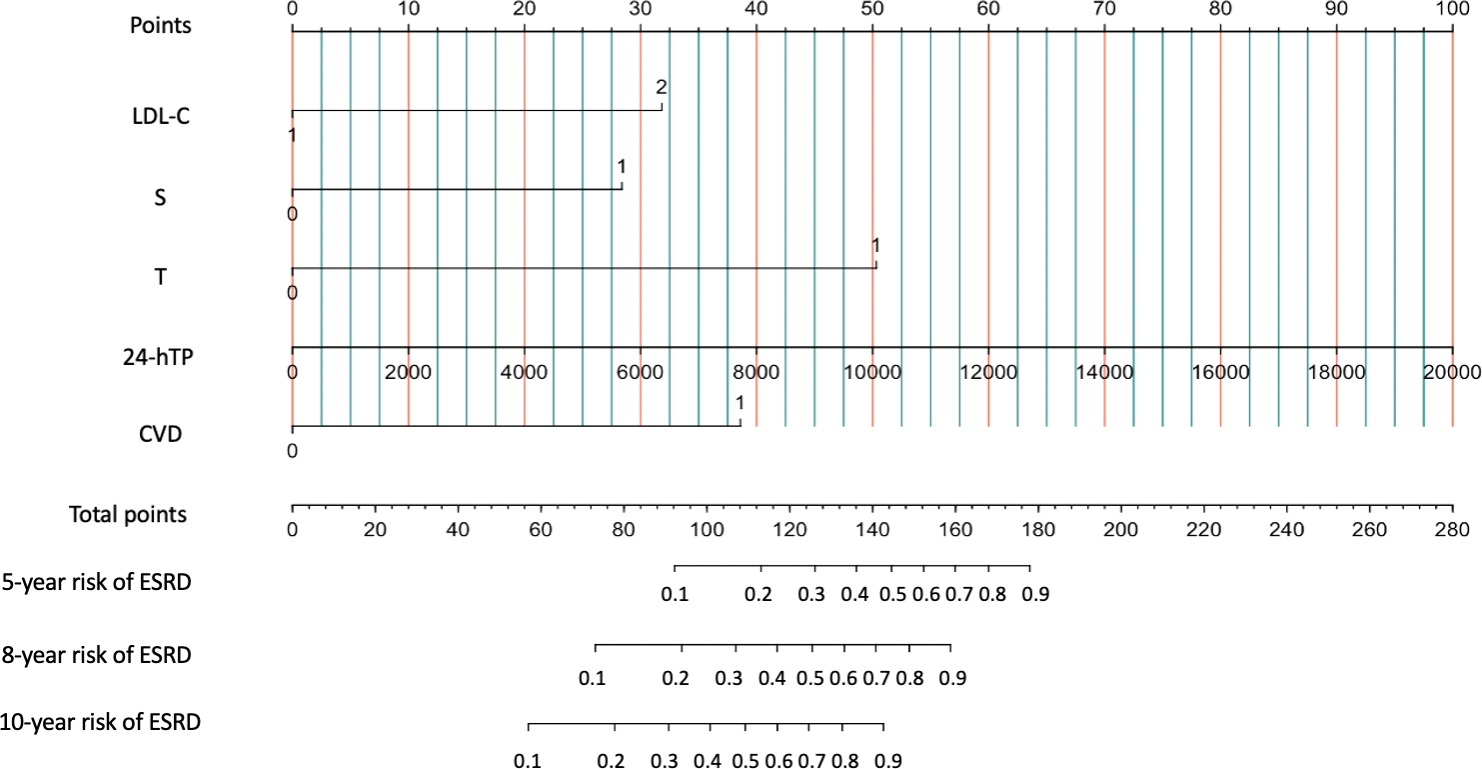
Nomogram predicts 5-, 8-, and 10-year risk of ESRD in IgAN. Calculation method: The value of each predictive parameter corresponds upward to the value on the Point axis, and then the “Point” values of all parameters are summed to correspond to the value on the “total point” axis, and downward to the 5-, 8-, and 10-year risk of ESRD in IgAN.

**Figure 4:**
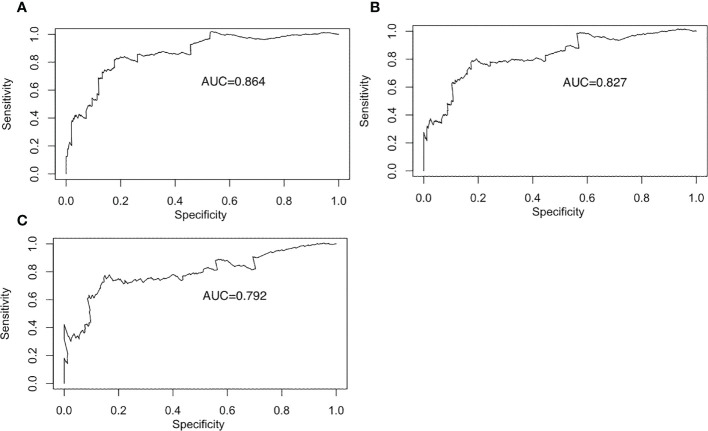
Receiver operating characteristic curve for the prediction model. Receiver operating characteristic curves for the 5-year prediction models. **(A)** Receiver operating characteristic curves for the 8-year prediction models. **(B)** Receiver operating characteristic curves for the 10-year prediction models. **(C)**.

**Figure 5:**
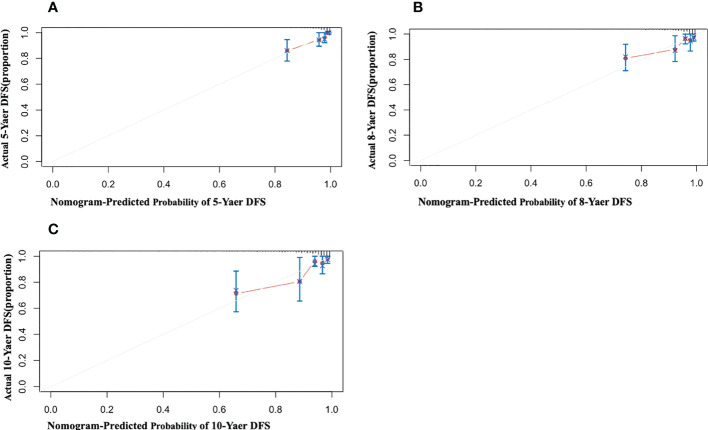
Calibration of the nomogram for Risk of ESRD in IgAN. The x-axis shows the predicted probability of risk of ESRD in IgAN, and the y-axis shows the observed probability of risk of ESRD in IgAN. **(A)** Nomogram of 5-year calibrated risk of ESRD in IgAN. C-index:0.862; **(B)** Nomogram of 8-year calibrated risk of ESRD in IgAN. C-index:0.838; **(C)** Nomogram of 10-year calibrated risk of ESRD in IgAN. C-index: 0.800.

When patients with LDL-C ≥ 2.60 mmol/L, they scored higher and indicate a worse prognosis. In addition, higher prevalence of CVD, S1, T1-2, 24-hour proteinuria and higher score of renal pathology indicated worse 5-, 8-, and 10-year risk of ESRD in IgAN (**Figure 3**). The AUC of the nomogram model was respectively evaluated at 0.864, 0.827, and 0.792 at 5, 8, and 10 years (**Figure 4**) and the corresponding C indices were respectively 0.862, 0.838, and 0.800, which indicated the nomogram model was accurate in predicting risk of ESRD in IgAN. The calibration against the nomogram model was also evaluated with the calibration curve (**Figure 5**) and the figure shows that the predictions are close to the observed results, which further demonstrates the reliability of the nomogram model in predicting risk of ESRD in IgAN.

## 4 Discussion

In this study, we found that IgAN patients with higher LDL-C level had severer clinical features, interstitial renal pathology changes and higher risk of ESRD. LDL-C, prevalence of CVD, S, T, 24-hour proteinuria were independent risk factors for prognosis of IgAN. Furthermore, we have developed a novel nomogram using these five indicators which showed valid and reliable prediction of 5-, 8-, and 10-year risk of ESRD in IgAN.

Dyslipidemia and renal function affect each. As renal function declines, a quantitative shift in lipid levels occurs which is characterized by elevated triglycerides, low HDL-C, and varying levels of ox-LDL and carbamylated LDL (c-LDL) cholesterol levels. Along with these quantitative changes, major qualitative changes in lipoprotein particles make them more atherogenic, including increased oxidation (15). Moorhead et al. suggested that dyslipidemia may be caused by loss of urinary protein and lead to compensatory reduction of lipoprotein synthesis and catabolism by the liver, which result in further renal injury (16). Tsutomu Hirano et al. proposed that renal insufficiency is linked to increased lipoprotein(a), and proteinuria is correlated with atherogenic subspecies of LDL (17). It has also been shown that hypercholesterolemia and hypertriglyceridemia are relevant to the deterioration of renal function in adults with IgAN (14). Our study found that IgAN patients with higher LDL-C level had worse renal function probably because patients in the high LDL-C group had higher oxLDL, which caused more oxidative stress resulting in glomerular and tubular damage, and thus was easier to enter ESRD.

Lipids are in close association with pathological impairment of IgAN. Hongjie Zhuang et al. showed that compared to children without dyslipidemia, children in the dyslipidemia group had severer clinical features and pathological changes, with higher proportions of S1 and C2 in the Oxford Classification of IgAN (18). Won Jung Choi et al. demonstrated that overall renal sclerosis, S, and M were higher in patients with IgAN in the high triglyceride group compared to the normal triglyceride group (19). This may be due to that dyslipidemia can cause renal tubular injury and promote renal fibrosis by inducing lipotoxicity, inflammation, oxidative stress and signaling events (20). Furthermore, the proximal tubule is rich in mitochondria, the main site of oxidative phosphorylation for energy supply, and is more susceptible to chronic inflammation and reactive oxygen species damage brought about by lipids (21). In addition, our study found that LDL-C was associated with IgA deposition, which may be related to the pathogenesis of IgAN and disease progression.

Some lipids are closely associated with the prognosis of IgAN. Studies have shown that high serum triglycerides are associated with a decrease in eGFR and a significant association with the incidence and progression of CKD (22). P Y Zuo et al. found that the ratio of NonHDLc/HDLc was an independent risk factor for the development of CKD and was useful in identifying people at high risk of CKD (23). After adjusting for potential confounders, patients in the highest tertile of NonHDLc/HDLc had a 1.45-fold higher risk of CKD than those in the lowest tertile (23). J Syrjänen et al. showed by developing a COX regression risk model that hypertriglyceridemia and hyperuricemia at diagnosis were adverse prognostic predictors for IgAN, whereas these factors were greatly underestimated previously (24). In our study, Kaplan-Meier analysis showed that IgAN patients in the high LDL-C group were more susceptible to ESRD. Additionally, multivariate COX regression analysis showed that LDL-C was an independent risk factor for IgAN patients. Our nomogram modeling showed higher scores in the high LDL-C group and higher 5-, 8-, and 10-year risk of ESRD in IgAN patients. Further, we firstly included prevalence of CVD, S, T and 24-hour proteinuria formation into the assessment parameters in combination with LDL-C to establish a nomogram prediction model and validated the reliability of the nomogram model by C-index and calibration plots.

Studies have shown that large amounts of filtered lipoproteins could contribute to the proliferation of mesangial cells, and the deposition of apolipoproteins in the renal tubules can lead to tubulointerstitial lesions (9). This provides a theoretical basis for the idea that lipid-lowering therapy can prevent renal fibrosis. However, some studies have shown that statin therapy modestly reduces the rate of proteinuria and eGFR decline, but not the incidence of renal endpoint events (25). The 2013 KDIGO guidelines recommend that non-dialysis CKD patients ≥50 years of age should be treated with statins or a statin/ezetimibe combination for lipid abnormalities, but do not specify therapeutic target values for lipids (5). In this study, we showed that the majority of IgAN patients were young adults around 33 years of age and in the early stages of CKD at the time of renal biopsy. However, LDL-C does represent an independent risk factor for the prognosis of IgAN and is more susceptible to ESRD when LDL-C ≥ 2.60 mmol/L. Therefore, the 2013 KDIGO guidelines may be too conservative in the use of lipid-lowering drugs for young IgAN patients in the early stages of CKD. Our study indiacted that when LDL-C is elevated above 2.60 mmol/L, clinicians should take a serious look at it and comprehensively evaluate the use of lipid-lowering drugs.

The present study also has some limitations. First, the exact mechanism of LDL-C involvement in IgAN pathogenesis is unclear and needs to be further explored. Second, the number of patients studied in this study was small and the study was a single-center cross-sectional study. Further prospective and cross-sectional studies could be conducted, including an expanded sample size, different ethnicities, regions, etc. Furthermore, the prediction model developed in this study needs to be corroborated by further external validation, as we only used internal validation.

In conclusion, elevated LDL-C level is a predictive factor for the prognosis of IgAN. We have developed and internally validated a novel nomogram which showed valid and reliable prediction of 5-, 8-, and 10-year risk of ESRD in IgAN.

## Data availability statement

The raw data supporting the conclusions of this article will be made available by the authors, without undue reservation.

## Ethics statement

This study was approved by the Ethics Committee of the Third Xiangya Hospital of Central South University (NO.22146). The patients/participants provided their written informed consent to participate in this study.

## Author contributions

Z-YT, A-ML wrote the first draft of the manuscript. HZ, LC, JH and XX contributed to conception and design of the study. All authors contributed to manuscript revision, read, and approved the submitted version.
